# National Trends in Radical Hysterectomy at Gynecologic Oncology Fellowship Training Programs

**DOI:** 10.1097/og9.0000000000000175

**Published:** 2026-05-28

**Authors:** Mitchel Hoffman, Dennis Chi, William Cliby, Karen H. Lu, Wenyi Fan, Daniel Clarke-Pearson

**Affiliations:** Department of Gynecologic Oncology, Moffitt Cancer Center and USF Morsani College of Medicine, Tampa, Florida; Department of Obstetrics & Gynecology, Weill Cornell Medical College, Gynecology Service, Department of Surgery, Memorial Sloan Kettering Cancer, New York, New York; Gynecologic Surgery, Mayo Clinic, Rochester, Minnesota; Moffitt Cancer Center, Houston, Texas; Biostatistics and Bioinformatics Shared Resource, Moffitt Cancer Center, Houston, Texas; and Department of Obstetrics and Gynecology, Division of Gynecologic Oncology, University of North Carolina School of Medicine, Chapel Hill, North Carolina.

## Abstract

**OBJECTIVE:**

To investigate trends in radical hysterectomy being performed by gynecologic oncology fellows in the United States.

**METHODS:**

Summary statistics for Accreditation Council for Graduate Medical Education (ACGME) programs were obtained from the ACGME public reporting system. For each academic year spanning 2019–2020 through 2024–2025, the national mean procedure count was extracted for radical hysterectomy.

**RESULTS:**

Over the past 5 years, there has been a significant decline in the number of radical hysterectomies being performed by fellows in the United States.

**CONCLUSION:**

Gynecologic oncology training number in radical hysterectomy has declined. This decline has implications that extend to training in other radical pelvic operations, including lymphadenectomy, cytoreductive surgery, and exenteration.

## Abstract 2

Gynecologic oncology training number in radical hysterectomy has declined.

Radical hysterectomy for early-stage cervical cancer was a common operation and the surgical foundation procedure for the development of gynecologic oncology as a subspecialty in 1972. The decline in cervical cancer incidence and refinement in treatment selection likely contributed to a reduction in the number of radical hysterectomies being performed across the United States.^1–3^ A 2019 publication by Hoffman et al^4^ reported a significant decline in the number of radical hysterectomies being performed at a single fellowship program over 20 years. CovelliVelez et al,^3^ using 2004 to 2020 data from the National Cancer Database and the Centers for Disease Control and Prevention's United States Cancer Statistics and published gynecologic oncology workforce data, reported that the annual rate of radical hysterectomies per gynecologic oncologist declined significantly, by an average of 6.9%/y, corresponding to a decrease from 4.5 to 1.5 cases per oncologist per year. Furthermore, retrospective and recent prospective studies have demonstrated that a significant subset of patients with early-stage cervical cancer may be safely managed with nonradical surgery.^5–8^ This evidence may have already changed practice dating back to the publication of these studies in 2021 and 2024 but will potentially continue to be more widely adopted and further diminish radical hysterectomy numbers at training centers.

The Accreditation Council for Graduate Medical Education (ACGME) first published surgery numbers for gynecologic oncology fellowships in the United States for the 2019–2020 academic year, with the most recent data available for the 2024–2025 academic year. These numbers are per graduating fellow (total experience during training) and are available through the ACGME website as public domain (last accessed April 21, 2026).^9^ This study evaluates the number of radical hysterectomies, based on ACGME data, performed by graduating gynecologic oncology fellows from 2019–2020 to 2024–2025.

## METHODS

This study is a repeated cross-sectional analysis of publicly reported, aggregated data from ACGME reporting system evaluating the national mean procedures across academic years from 2019–2020 to 2024–2025. Summary statistics for ACGME programs were obtained from the ACGME public reporting system (https://apps.acgme.org/ads/Public/Reports/Report/25). Data were restricted to the specialty of gynecologic oncology (specialty code 225) and academic years spanning 2019–2020 through 2024–2025. For each academic year, the national average, SD and number of fellows, and percentile distributions of case volumes (10th, 30th, 50th, 70th, and 90th percentiles) were extracted for radical hysterectomy. Trends over time were evaluated with inverse-variance weighted linear regression applied to yearly summary statistics. For each year, the average of procedures and its associated SD and sample size of fellows were used to estimate the variance of the mean. Linear regression models were then fitted with year as a continuous predictor and weighted by the inverse of the estimated variance such that years with more precise estimates contributed greater weight to the analysis. The statistical significance of trends was assessed with the *P* value for the slope coefficient from the weighted regression model. All analyses were performed with R 4.5.2.

## RESULTS

**Fig. 1.**
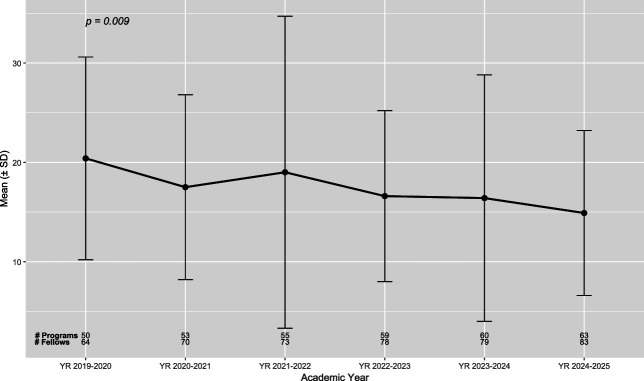
Accreditation Council for Graduate Medical Education 2019–2025 linear trend, all roles, radical hysterectomy.

The number of total programs and fellows was observed in increasing trend from 2019 to 2025 (Fig. 1 and Tables 1 and 2). Five-year trends in the recorded number of radical hysterectomies performed by graduating gynecologic oncology fellows are shown in Figure 1. We observed a progressive downward trend (β=−0.92, 95% CI, −1.46 to −0.38) (*P*<.05) in the reported mean number of radical hysterectomies. There was substantial variation in reported fellow experience (Tables 1 and 2). Fellow numbers in the 50th percentile for the 2019–2021 academic year (17 cases) were comparable to those in the 70th percentile cases for the 2024–2025 years (17 cases) (Tables 1 and 2). Fellow numbers in the 10th percentile for the 2024–2025 academic year (six cases) were below the newly proposed minimums set by the ACGME (10 cases).

*Hoffman. Trends in Radical Hysterectomy. O&G Open 2026*.

**Table 1.** Gynecologic Oncology Fellowship Case Log Data for U.S. Fellows Graduating From 2019 to 2020

Total Experience 2019–2020 Among 64 Fellows					
Percentile	10th	30th	50th	70th	90th
Radical hysterectomy (n)	8	14	17	22	32

Source: https://apps.acgme.org/ads/Public/Reports/Report/25.

**Table 2.** Gynecologic Oncology Fellowship Case Log Data for U.S. Fellows Graduating From 2024 to 2025

Total Experience 2024–2025 Among 83 Fellows					
Percentile	10th	30th	50th	70th	90th
Radical hysterectomy (n)	6	10	13	17	27

Source: https://apps.acgme.org/ads/Public/Reports/Report/25.

## DISCUSSION

Fellow experience in performing radical hysterectomy has decreased significantly since 2019. The ACGME has recently established minimum targets for graduating fellows, and our observations suggest that some programs may struggle to meet the minimum. In addition, we observed meaningful variations in experience across fellowships, reaffirming the need for ACGME minimums. It must be noted, however, that these minimums have been set for the first time for gynecologic oncology, effective for the 2025–2026 academic year. The plan by ACGME is that these minimums will not be enforced until review of the 2027 graduate data. Regardless, the reduction in this procedure raises questions about how to maintain critical surgical skills.

It is noteworthy that the ACGME data also indicate a substantial decline in some of the other radical procedures, including pelvic and paraaortic lymphadenectomy. A decline in surgical volume and a reduced preparedness for fellowship training have been noted across obstetrics–gynecology as a specialty.^10,11^

The present findings are consistent with a prior report of long-term trends (2001–2019 graduates) in fellow surgical procedures reported from a subset (n=14) of long-standing gynecologic oncology fellowship programs.^12^ The median number of radical hysterectomies performed ranged from approximately 17 to 32 throughout the 18-year period, similar to the median number of 20 in the 2019–2020 ACGME database. Our current findings document the subsequent decline of radical hysterectomy coincident with changes in practice. In addition, we observed significant variation in the reported fellow experience in these procedures nationally. The recent ACGME announcement of proposed minimums for the first time is a welcome first step in addressing surgical training. The proposed minimum for radical hysterectomy is 10. Although we did not have access to minimum numbers, the data available indicate that most fellowship programs would have met the ACGME requirement. However, there is a wide SD around the mean, suggesting that some programs provide less surgical experience compared with others. As with any surgical procedure, the number of cases performed may not confirm competence. For example, the learning curve to define competence in performing a total laparoscopic hysterectomy has been reported to be approximately 50 cases.^13–15^ A future goal of training and certification should be heightened emphasis on measures of competency and not simply meeting minimum numbers. Having one or more experienced surgical educators sign off on a trainee, based on direct observation, as competent to perform a procedure is likely more meaningful than documenting the performance of a prespecified number of that procedure.

Simplified means of treating early-stage cervical cancer (nonradical surgery, refined methods of radiotherapy) will likely continue to reduce the indications for radical hysterectomy. It will be increasingly difficult to train and maintain the skill set necessary to perform this operation. It is not possible to determine what percentage of radical hysterectomies currently being performed might be appropriately managed with nonradical surgery. To the best of our knowledge, data indicating what percentage of radical hysterectomies currently being performed might be managed with nonradical surgery have not been published. The ACGME data do not provide specific clinical information that would give insight into this percentage. Moreover, there is overlap between the recent studies mentioned^7,8^ and the National Comprehensive Cancer Network guidelines.^16^

Radical hysterectomy has been central to surgical training in gynecologic oncology. Several important techniques are learned from this operation that carry over to the broader spectrum of radical female pelvic surgery, including development of retroperitoneal spaces, pelvic ureterolysis, surgical management of the paracervical and paravaginal ligaments, and pelvic lymphadenectomy. The reduction in experience with radical hysterectomy has likely had a significant, albeit difficult to define, negative effect on overall surgical training.

Evolution in surgical training should occur to ensure adequate experience in performing radical hysterectomy and the varied surgical skills that accompany it. Options include restructuring current training and developing novel training models and continuing medical education programs (ie, hands-on courses, mentoring, coaching). The range of tools from simulation to cadaveric and animal models is beyond the scope of this article. However, this is an opportunity for our professional societies and accreditation and certification organizations to work collectively to enhance the training and continued development of the specialty of gynecologic oncology. Moving from procedural numbers to assessment of competency in training would be valuable.

The strength of the current study is that it used national data from ACGME fellowship programs in the United States. The study has several limitations. Data are available only for 6 recent years. Given the declining incidence of cervical cancer, it is likely that the declining trend in the number of radical hysterectomies began before this. The analyses relied exclusively on publicly available summary statistics rather than individual program-level or trainee-level data. In addition, both fellows and programs were expanding during the reporting period and becoming familiar with reporting requirements, so some underreporting may have occurred, which is not possible to measure. Trend analyses were based on inverse-variance weighted linear regression of yearly means and were subject to assumptions of normality and independence. These analyses do not account for within-year heterogeneity or individual-level confounding, and findings should be interpreted as descriptive of overall temporal trends.

This study demonstrates that current gynecologic oncology fellows are completing training with fewer radical hysterectomies than were performed even 5 years previously. Much of this decline is a result of advances in surgical research that supported treatment with nonradical surgery: these changes do not remove the need for full dissection in some cases. The challenge for our specialty is how to fill the gaps in acquiring surgical expertise brought about by changes in clinical practice.
